# A Case of Bilateral Thalamic Infarcts Involving the Artery of Percheron in the Setting of COVID-19

**DOI:** 10.7759/cureus.15587

**Published:** 2021-06-11

**Authors:** Branden Wilson, Aswin Srinivasan, Tusharkumar Pansuriya, Salman Alim, Uzma Ali

**Affiliations:** 1 Internal Medicine, Hospital Corporation of America (HCA) Houston Kingwood/University of Houston College of Medicine, Houston, USA; 2 Internal Medicine, Hospital Corporation of America (HCA) Houston Kingwood/University of Houston College of Medicine, Kingwood, USA; 3 Pulmonary and Critical Care Medicine, Hospital Corporation of America (HCA) Houston Kingwood/University of Houston College of Medicine, Kingwood, USA; 4 Neurology, Hospital Corporation of America (HCA) Houston Kingwood/University of Houston College of Medicine, Kingwood, USA

**Keywords:** artery of percheron, stroke, thalamic stroke, covid-19, bilateral thalamic stroke, atypical stroke, covid-19 associated coagulopathy, rt-tpa, stroke and covid-19, altered mental status evaluation

## Abstract

The artery of Percheron (AOP) is a rare anatomic variant, characterized by a single thalamoperforating artery arising from the P1 segment of the posterior cerebral artery that bifurcates to supply bilateral thalami with variable vascular supply to the midbrain. The occlusion of this artery is responsible for bilateral thalamic stroke with or without midbrain involvement. Since December 2019, coronavirus disease 2019 (COVID-19) caused by severe acute respiratory syndrome coronavirus 2 (SARS-CoV-2) has led to a worldwide pandemic. Acute ischemic stroke is a rare but known manifestation of COVID-19. There have only been a few reports of bilateral deep cerebral involvement in COVID-19 infection. In the absence of risk factors for such events, we suspect COVID-19 may have a contributory role. In this case report, we present a case of AOP infarction presenting as transient loss of consciousness, intermittent anisocoria, dysarthria, and right-sided weakness in the setting of COVID-19 infection. Given the degree of variation in clinical presentation for AOP infarcts and lack of evidence of ischemia on initial imaging studies, many patients may miss the time window for tissue plasminogen activator (tPA) administration. This case highlights the importance of timely neurological evaluation in patients presenting with COVID-19 and neurological complaints. Increased community awareness of neurological manifestations of AOP infarctions is of utmost importance as early detection and intervention improve clinical outcomes.

## Introduction

The thalamus is a complex paired gray matter structure located in the forebrain. It is important for many critical functions of the brain including relaying ascending sensory information between different subcortical areas and the cerebral cortex. Its vascular supply is predominantly supplied by perforating branches of P1 and P2 segments of the posterior cerebral artery (PCA) and posterior communicating arteries [1,2]. The thalamic vascular supply can be categorized into 4 territories: anterior, paramedian, posterior, and inferolateral [1,2]. Specifically, the paramedian territory of the thalamus vascular supply is supplied by the paramedian arteries (thalamoperforating arteries), which arise from the P1 segment of the PCA [1,2]. The paramedian arteries have been reported to have variant forms with respect to vascular supply to the thalamus and midbrain [1,2]. One rare anatomical variant is the artery of Percheron (AOP), characterized by a single thalamoperforating artery arising from the P1 segment of the PCA that bifurcates to supply bilateral paramedian thalami with variable vascular supply to the midbrain [1-3]. Ischemic patterns that result from occlusion of AOP show bilateral paramedian thalamic infarcts with or without midbrain involvement [1-4]. The clinical presentation of AOP ischemic infarction varies considerably due to the comprehensive role of critical functions controlled by the thalamus, making diagnosis challenging for the clinician [1-4].

Since December 2019, coronavirus disease 2019 (COVID-19) caused by severe acute respiratory syndrome coronavirus 2 (SARS-CoV-2) has led to a worldwide pandemic [5]. Respiratory and gastrointestinal systems are commonly affected by this infection. Neurological manifestations such as acute ischemic stroke associated with a hypercoagulable state caused by COVID-19 are increasingly being reported [6]. In this case report, we present a unique case of AOP infarction presenting as transient loss of consciousness, intermittent anisocoria, dysarthria, and right-sided weakness in the setting of COVID-19 infection. To our knowledge, AOP infarction associated with concomitant COVID-19 infection has not been reported in the literature.

## Case presentation

A 56-year-old male with a past medical history of hypertension and obstructive sleep apnea (OSA) was found unconscious by his wife. He was last known in his usual state of health by his wife after waking at 6:30 am on the day of presentation. Approximately two hours later, he complained of dizziness and was noticed to have difficulty walking. A few minutes later, his wife found him unresponsive lying on his back. Emergency Medical Services (EMS) was called and the patient was air-lifted to our emergency department (ED) arriving at 09:18 am. On arrival to the ED, he was afebrile, his blood pressure was 156/87 mmHg, heart rate was 87 beats per minute, respiratory rate was 14 breaths per minute, and oxygen saturation was 94% on three liters of oxygen. On exam, he had asymmetric pupils and would only withdraw to pain in the left upper extremity and left lower extremity. He had no response to pain in the right upper extremity and had a weak response in the right lower extremity. Subsequently a few minutes after arrival, he awoke with dysarthria and continued to have persistent right-sided weakness in his upper and lower extremities. His National Institute of Health Stroke Scale (NIHSS) was 24. Computer tomography (CT) of head/brain without contrast performed at 09:25 am did not reveal any acute intracranial abnormality. CT angiography of head/neck performed at 09:28 am was unremarkable for any dissection, stenosis, occlusion, or aneurysm. Due to persistent deficits seen on exam and concern for an acute ischemic stroke, he was given a tissue plasminogen activator (tPA) at 09:53 am. There were also concerns for seizure, so the patient was loaded with 1 g of levetiracetam.

**Figure 1:**
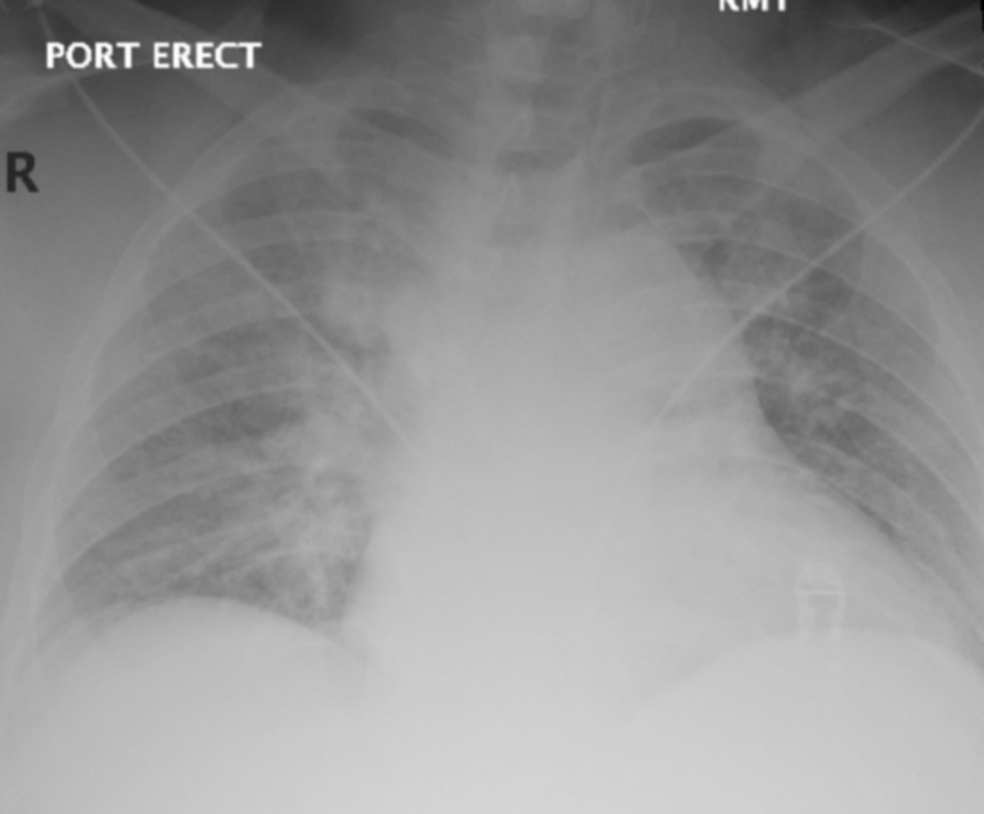
Anteroposterior (AP) chest x-ray Single frontal view of the chest showing interstitial and alveolar infiltrates in both lungs

Laboratory studies, such as serum sodium, serum potassium, blood urea and nitrogen, serum creatinine were within normal limits. Serum CRP level was 8.5 mg/L (normal range 0-9 mg/L). D-dimer was elevated at 851 ng/mL FEU (normal range 0-500 ng/mL FEU), prothrombin time (PT) was 12.0 s (normal range 9.2-12.1 s) with an international normalized ratio (INR) of 1.0, partial thromboplastin time (PTT) was 23.5 s (normal range 23.4-37.0 s). His lipid panel was within normal limits. Hemoglobin A1C was 5.0 (normal range 0-5.9). He tested reverse transcriptase-polymerase chain reaction (RT-PCR) positive for SARS-CoV-2. Initial chest x-ray showed increased interstitial and alveolar opacities in both lungs (Figure 1). His oxygen requirements continued to increase in the emergency department so non-invasive ventilation with bilevel positive airway pressure (BiPAP) was started. His COVID-19 pneumonia was treated with intravenous antibiotics, remdesivir, and steroids. He was admitted to the Intensive care unit (ICU) for close monitoring.

**Figure 2:**
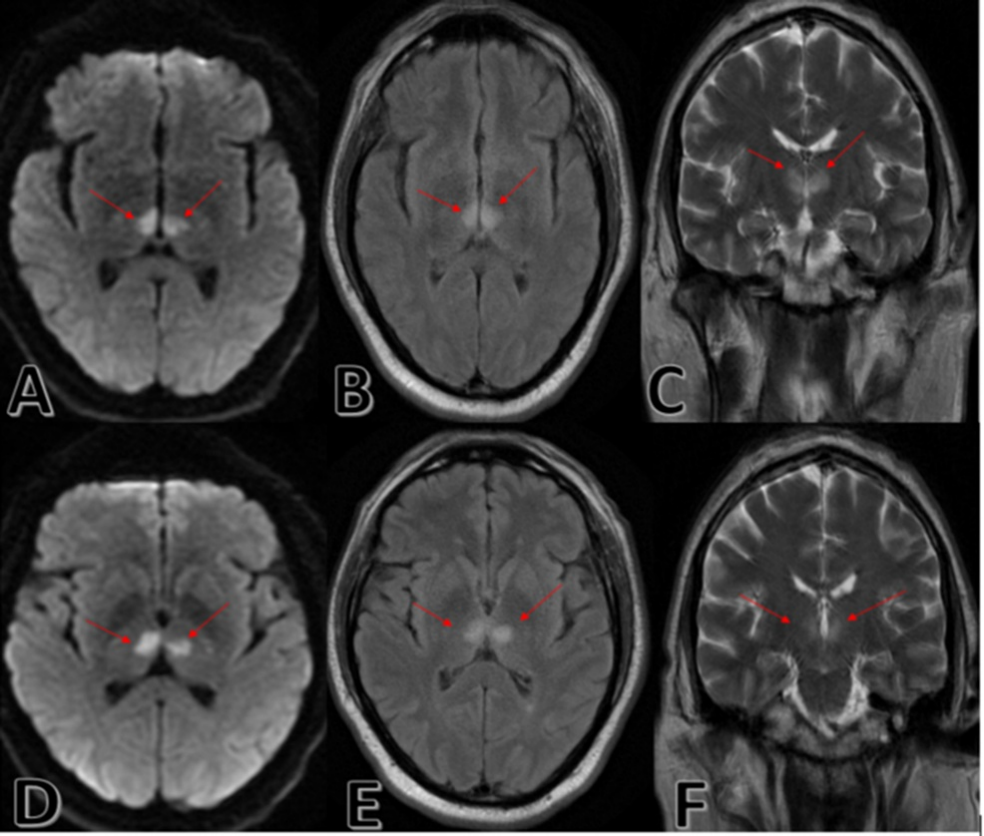
Bilateral thalamic infarcts suggestive of stroke from occlusion of AOP (A, D) Axial DWI MRI view, showing bilateral paramedian thalamic restricted diffusion in the area supplied by AOP. (B, E) Axial T2 FLAIR MRI showing bilateral thalamic restricted diffusion in the area supplied by AOP. (C, F) Coronal section T2 FLAIR sequence showing bilateral thalamic infarct. DWI: Diffusion-weighted imaging; FLAIR: Fluid-attenuated inversion recovery; MRI: Magnetic resonance imaging; AOP: Artery of Percheron

**Figure 3:**
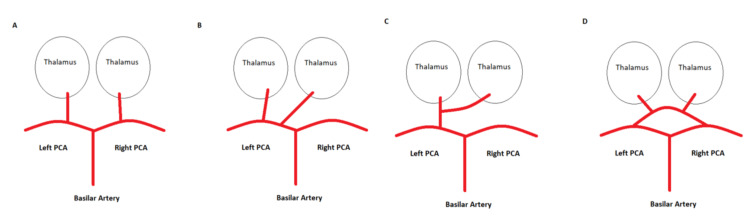
Paramedian thalamic artery variants (A) Type I, normal anatomy. (B) Type IIa, both paramedian arteries originate from a single P1 segment. (C) Type IIb, the AOP originates from a single main trunk from the P1 segment, supplying the bilateral paramedian thalamus and midbrain regions. (D) Type III, an arterial arcade connects the left and right P1 segments of both PCAs which give rise to the paramedian arteries. AOP: Artery of Percheron; PCA: Posterior cerebral artery; P1: Segment one of the posterior cerebral artery

The following day his focal neurological deficits were much improved. He was alert, awake, and oriented and following commands appropriately. His face was symmetric and tongue protrusion was midline. His speech was moderately dysarthric and he had complete vertical gaze palsy on the exam. He was moving all extremities with deep tendon reflexes of 3+ with clonus in bilateral lower extremities and 2+ in upper extremities. The sensation was subjectively intact to light touch on both sides. Coordination was intact to rapid alternating movements in the fingers as well as finger-nose-finger testing. As his mental status and oxygen requirements improved, he was downgraded from the ICU. Magnetic resonance imaging (MRI) of the brain without contrast revealed restricted diffusion involving bilateral medial thalami on diffusion-weighted imaging (DWI) suggestive of acute infarcts (Figures 2A-2F). This was thought to be secondary to variant vascular anatomy, namely, the AOP (Figures 3A-3D). Therapeutic enoxaparin was added for anticoagulation and later transitioned to apixaban. Electroencephalogram (EEG) showed mild encephalopathy but did not reveal any clear, focal, lateralizing or epileptiform activity. CT venogram was negative for dural venous sinus thrombosis. Transthoracic echocardiography (TTE) was unremarkable, which ruled out patent foramen ovale (PFO) as a potential etiology.

On day 4 of his hospitalization, his symptoms were improved significantly. He was safely discharged home with outpatient stroke rehabilitation for his dysarthria and vertical gaze palsy. During his one-month follow-up visit with neurology, his dysarthria had mildly improved but he still suffered from complete vertical gaze palsy. He was referred to neuro-ophthalmology for vertical gaze palsy.

## Discussion

Ischemic stroke is a rare but known manifestation of COVID-19 [6]. One large retrospective study shows that the incidence of the stroke in COVID-19 patients is slightly more than 1%, but cannot be ignored [7]. Recent studies have demonstrated that patients who are COVID-19 positive were more likely to have a cryptogenic cause of stroke when compared to COVID-19 negative patients, suggesting an association of COVID-19 disease with stroke [6,7]. This association of COVID-19 disease with acute ischemic stroke is likely secondary to multiple factors as SARS CoV-2 has been postulated to cause endothelial damage, severe cytokine release, generalized hypercoagulability, as well as direct cytopathic effect on the central nervous system [6-8]. Hypercoagulability is one of the features of pathogenesis of COVID-19 disease [8].

In a recent systematic review of COVID-19 and cerebrovascular events, Frisullo et al. reported different stroke profiles based on serum markers for systemic inflammation and coagulopathy, stroke type, and vascular territory [9]. Frisullo et al. identified a cerebral small vessel profile of patients who developed acute stroke with COVID-19 symptoms simultaneously [9]. This group was found to have a low degree of disability with low serum markers for systemic inflammation and coagulopathy [9]. Interestingly, our patient fits this stroke profile as he developed an acute stroke simultaneously with COVID-19 symptoms. He was also found to have low systemic inflammatory and prothrombotic serum markers. SARS-CoV-2 infection is mediated by angiotensin-converting enzyme 2 (ACE2) receptors, which are expressed on the epithelium of the small intestine, kidney, and lung. ACE2 receptors are also abundant on vascular endothelium and arterial smooth muscle cells [9-12]. The direct local effect of SARS-CoV-2 on ACE2 receptors in the vascular endothelium has been reported to induce an inflammatory response that has been postulated to be a constituent of thrombotic events associated with infection [10-12]. In one post-mortem analysis of patients infected with SARS-CoV-2, Varga et al. reported signs of endotheliitis with the presence of viral elements within endothelial cells and accumulation of inflammatory cells, suggesting an important role of endothelial cell injury in patients with COVID-19 [10]. SARS-CoV-2-related microangiopathic damage secondary to either direct viral damage or a local immune response could explain cerebrovascular events in patients without increased systemic inflammatory or prothrombotic serum markers.

Other rare cerebrovascular events associated with COVID-19 infection as well as bilateral deep cerebral structural involvement have been reported in the literature [13-15]. Daci et al. reported a case of a patient with COVID-19 who was found to have bilateral basal ganglia hemorrhage [13]. Bilateral basal ganglia hemorrhage is a rare phenomenon and in the absence of risk factors for such an event, COVID-19 infection may have a contributory role. In the systematic review described by Frisullo et al., an intracranial bleeding profile was identified, characterized by a high disability and poor outcome in patients with few cerebrovascular risk factors. Serum markers of systemic inflammation and coagulopathy were likely to be normal in this group [9]. Direct viral damage via ACE2 receptors on vascular endothelium and arterial smooth muscle cells in intracranial arteries leading to vessel wall rupture could explain such phenomenon [9-12]. Poyiadji et al. reported a rare case of a woman with COVID-19 who presented with a three-day history of cough, fever, and altered mental status who was found to have hemorrhagic rim-enhancing lesions within the bilateral thalami and medial temporal lobes [14]. She was presumed to have COVID-19-associated acute necrotizing hemorrhagic encephalopathy and was treated with intravenous immunoglobulin. Acute necrotizing encephalopathy is a rare complication of viral infections associated with intracranial cytokine storms resulting in blood-brain barrier breakdown [14]. This could lead to a parenchymal brain hemorrhage [11,14]. Tiwari et al. reported a case of a 9-year-old infant with COVID-19-associated multisystem inflammatory syndrome who was found to have multifocal discrete and confluent hypodensities suggestive of infarcts in the genu and adjacent body of corpus callosum, left basal ganglia, and bilateral thalami [15]. The SARS-CoV-2 infection leads to the production of pro-inflammatory cytokines via T-helper type 1 and 2 reactions [9,11,12]. IL-6 is one key mediator that plays a crucial role in inflammation of SARS-CoV-2 infection [9,11,12]. IL-6 induces the synthesis of CRP, a known proinflammatory marker in atherothrombotic vascular disease and stroke [9,11,12]. Other proinflammatory cytokines induced by SARS-CoV-2 infection upregulate procoagulants as well as downregulate anticoagulants contributing to a state of hypercoagulability [11,12]. This immune response produces a systemic immune-mediated disease causing a more severe COVID-19 infection that may result in acute stroke [9,11,12]. Antiphospholipid antibodies and lupus anticoagulants have been reported in COVID-19 patients with multiple hemispheric infarcts [9,11,12]. Interestingly, these patients are also commonly found to have concomitant elevation in serum markers for hypercoagulability as well as thrombocytopenia [9,11,12].

These rare cerebrovascular events in patients with COVID-19 infection are likely secondary to multiple factors as described above. Measuring systemic inflammatory (CRP, IL-6) and coagulopathy markers (D-dimer, PT/INR, PTT, Fibrinogen) can assist in recognizing signs of severe systemic inflammatory and hypercoagulability responses associated with these rare events. Antiphospholipid antibodies and lupus anticoagulants when indicated could also prove helpful. Further evidence is needed from larger studies to confirm the relationship between these rare events and the pathologic mechanism of SARS-CoV-2 infection.

Our case showed a bilateral thalamic stroke in association with COVID-19 disease with normal systemic inflammatory markers. This decreases the likelihood of a systemic inflammatory cause of stroke in our patient. AOP is a rare anatomic variant of the blood supply of the thalamus. The occlusion of this artery is responsible for the bilateral thalamic stroke. Bilateral thalamic stroke is a rare phenomenon, accounting for only 0.1% to 2% of all ischemic strokes worldwide [1-4]. We postulate that a virus-induced state of impaired microcirculatory function secondary to direct viral damage of vascular endothelium with local immune dysregulation in the brain could have resulted in our patient's acute stroke. Our patient's presentation with transient loss of consciousness, intermittent anisocoria, dysarthria, and right-sided weakness is one of the few described in the literature for AOP infarcts. The presentation of fluctuating anisocoria and vertical gaze palsy can often be misdiagnosed for seizure instead of stroke. This has important implications regarding the management as it affects the time window for tPA administration. Early detection and treatment with tPA significantly improve morbidity and mortality in these patients. Due to prompt recognition of this presentation of thalamic stroke in the COVID-19 setting, tPA was administered which resulted in improvement of some of the focal neurological findings on the same day. Given the degree of variation in clinical presentation for AOP infarcts and lack of evidence of ischemia on initial imaging studies, many patients may miss the time window for tPA administration.

## Conclusions

We present a unique case of bilateral thalamic stroke secondary to AOP in a patient with COVID-19 infection. To our knowledge, AOP infarction in the setting of COVID-19 infection has not been reported in the literature. A few rare cases of bilateral deep cerebral involvement in the setting of COVID-19 infection have been reported in the literature. In the absence of risk factors for such events, SARS-CoV-2 may play a contributory role. This case highlights the importance of timely neurological evaluation in patients presenting with COVID-19 and neurological complaints. It is important to keep AOP infarction in the differential diagnosis for neurological presentations such as vertical gaze palsy and anisocoria, as early detection and intervention can improve clinical outcomes. As our understanding of COVID-19 and its clinical manifestations continue to evolve, clinicians need to be prepared to encounter and recognize atypical extra-pulmonary manifestations of COVID-19 infection.
